# Uncovering the essential links in online commercial networks

**DOI:** 10.1038/srep34292

**Published:** 2016-09-29

**Authors:** Wei Zeng, Meiling Fang, Junming Shao, Mingsheng Shang

**Affiliations:** 1 Trusted Computing and Automated Reasoning Lab, University of Electronic Science and Technology of China, Chengdu 611731, P. R. China; 2 Web Sciences Center, University of Electronic Science and Technology of China, Chengdu 611731, P. R. China; 3 Big Data Research Center, University of Electronic Science and Technology of China, Chengdu, China; 4 Chongqing Institute of Green and Intelligent Technology, Chinese Academy of Sciences, Chongqing 400714, P. R. China

## Abstract

Recommender systems are designed to effectively support individuals' decision-making process on various web sites. It can be naturally represented by a user-object bipartite network, where a link indicates that a user has collected an object. Recently, research on the information backbone has attracted researchers' interests, which is a sub-network with fewer nodes and links but carrying most of the relevant information. With the backbone, a system can generate satisfactory recommenda- tions while saving much computing resource. In this paper, we propose an enhanced topology-aware method to extract the information backbone in the bipartite network mainly based on the information of neighboring users and objects. Our backbone extraction method enables the recommender systems achieve more than 90% of the accuracy of the top-L recommendation, however, consuming only 20% links. The experimental results show that our method outperforms the alternative backbone extraction methods. Moreover, the structure of the information backbone is studied in detail. Finally, we highlight that the information backbone is one of the most important properties of the bipartite network, with which one can significantly improve the efficiency of the recommender system.

Recently, recommender systems have been widely studied and designed to assist individuals in finding their interested contents such as books, movies and music. So far, many recommendation algorithms have been proposed, such as collaborative filtering (CF)^1^, matrix factorization^2^, latent sematic indexing^3^, multi-dimensional scaling^4^ and so on. In addition, a recommender system can be represented by a user-object bipartite network, where a node denotes a user or an object and a link indicates that a user has collected an object. By relying on the network topology, some network propagation processes such as the mass diffusion^5^,^6^ and the heat conduction^7^, have been applied in the recommender system. Researchers also find that the hybridization of these two methods can solve the accuracy-diversity dilemma in the recommender system^8^. More recently, a semi-local diffusion method is proposed to solve the sparsity problem in the recommender system^9^.

One of the most important challenges in the research on recommender system is how to improve the efficiency of a recommendation algorithm. It is of great importance for a recommender system to generate instant object recommendations for individual users to keep their loyalty. In the recent, some network manipulation methods are proposed to address the problem. Zeng^10^ studied the relevance of users in a recommender system and found that there exists a small group of key users. With them, the recommender system can generate satisfactory recommendations. That is, not all the information in the user-object bipartite is necessary when recommending objects for users. Similar conclusion can be found in ref. 11, which reveals that the performance of recommendation algorithms can be improved by eliminating some redundant links in the network. The authors proposed time-aware and topology-aware link removal algorithms to extract the information backbone in a recommender system.

The backbone is one of the most important properties of the complex network^12^,^13^. It is a sub-network with fewer links and nodes but preserving the topological properties or the function of the original network such as the degree distribution^14^, transportation ability^15^ and betweenness^16^. In the user-object bipartite network, an information backbone is defined as a sub-network with a group of users, a set of objects and their links, which carries most of the relevant information for object recommendations. Only relying on the information backbone, a recommender system’s efficiency can be greatly improved.

Although some methods are proposed to uncover the information backbone, there are still some problems left. For instance, most of these methods are utilized in unipartite networks^13^,^16^,^17^,^18^. Few of them can be used in bipartite networks. Moreover, the information of neighboring users and objects is not considered for the topology-aware backbone and the evolution of the network is not taken into account for the time-aware backbone^11^. In this work, we propose a topology-aware backbone extraction method which exploits the structure of the subgraph consisting of neighboring users and objects. After verification on three real-world bipartite networks, the obtained results show that our method performs better than the alternative backbone extraction techniques. Moreover, structure properties of the information backbone are analyzed in detail.

## Results

A recommender system can be naturally denoted by a user-object bipartite network *G*(*U*, *O*, *E*), where *U* = {*u*_1_, *u*_2_, …, *u*_*n*_}, *O* = {*o*_1_, *o*_2_, …, *o*_*m*_} and *E* = {*e*_1_, *e*_2_, …, *e*_*l*_} are sets of users, objects and links, respectively. It can also be represented by an adjacency matrix *A* = {*a*_*iα*_}, where the element *a*_*iα*_ = 1 if user *i* has collected object *α*, and 0 otherwise (We use Greek and Latin letters, respectively, for object-related and user-related indices). For a target user to whom we will recommend objects, each of her uncollected objects will be assigned a score by the recommendation algorithm and the top-*L* objects with the highest scores will be recommended. Different algorithms generate different object scores and thus different recommendation lists for users.

**Figure 1:**
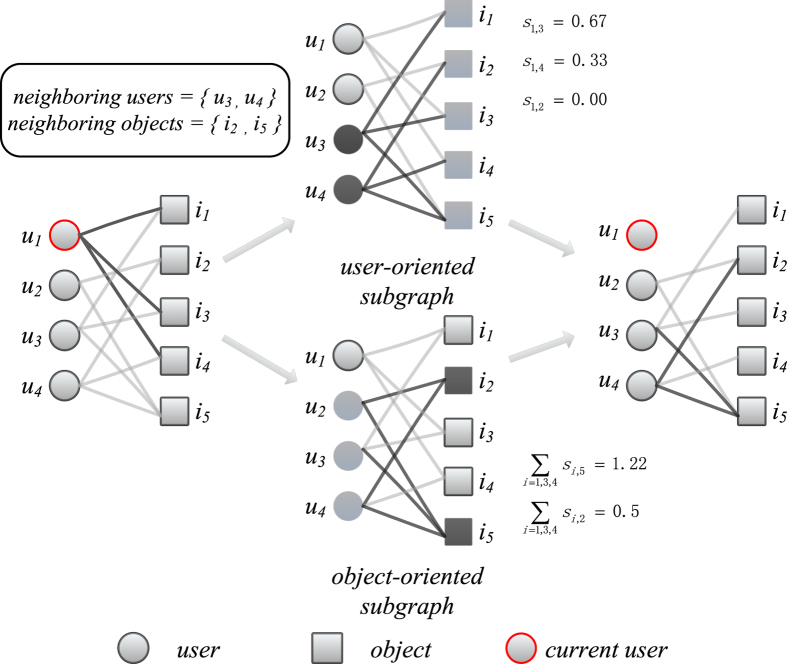
A visualization of the subgraph-based method. Given user *u*_1_, the user-oriented and object-oriented subgraph are constructed by relying on *u*_1_’s neighboring users and objects.

To the best of our knowledge, some methods are proposed to uncover the information backbone in a bipartite network. The first one is simply based on the popularity of links, with the underlying hypothesis that the relevance of a link can be reflected by its popularity (Popularity for short). The popularity of link *e*_*iα*_ is defined as *k*_*i*_*k*_*α*_, where *k*_*i*_ and *k*_*α*_ are the degree of user *i* and object *α*, respectively. The information backbone thus consists of links with the largest popularity. The second one is to randomly select a set of links as the information backbone. This method is used as a benchmark for comparison (Random for short). In the third method, the frequency of rectangles that each link belongs to is utilized to measure the relevance of links. A rectangle is defined as a subgraph consisting of a pair of users and objects. Those links which appear most frequently are selected as the information backbone (Rectangle for short). As mentioned above, these methods didn’t take into account the information of neighboring users and objects, which may make a recommendation algorithm inaccurate. In this paper, we propose a subgraph-based method to uncover the information backbone, which computes the frequency of user-oriented and object-oriented subgraphs that each link belongs to. For user *i*, we can get her *K* nearest neighbors by ranking the cosine similarity: 
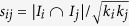
, where *I*_*i*_ is the set of objects collected by user *i*. Those neighboring users and their collected objects make up a subgraph. Likewise, we can also obtain a set of objects which are the most similar to *i*’s collected objects. Each of *i*’s uncollected objects will be assigned a score: 
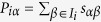
, where 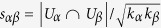 is the similarity between object *α* and *β*. Those objects with highest scores and their links can also comprise a subgraph. We then count the frequency that a link belongs to these two kinds of subgraphs. By repeating the above process over all users, we can get the final frequency of each link and those links with the largest frequency will be chosen as the information backbone (Subgraph for short). A visual representation of the subgraph-based method is given in Fig. 1.

Once we get the information backbone, only the links in the backbone are used to run the recommendation algorithm. We then compare the performance of the recommendation algorithm relying on the backbone with those relying on the original network. Tests are executed between our information backbone extraction method and four recommendation algorithms (see Methods), namely mass diffusion (MD for short)^5^, mass diffusion and heat conduction hybrid method (Hybrid for short)^8^, User-based collaborative filtering (UCF for short)^1^ and Item-based collaborative filtering(ICF for short)^19^.

We run these four recommendation algorithms on the original bipartite network and the information backbone, respectively. For those users who have no links in the backbone, we recommend the most popular objects as a compromise. In order to evaluate the performance of our methods, we utilize three data sets in this paper, i.e. Douban, Flixster and Flickr (see Table 1). Precision as well as Ranking score are computed to evaluate the performance of recommendation algorithms (see Methods and SI).

**Figure 2:**
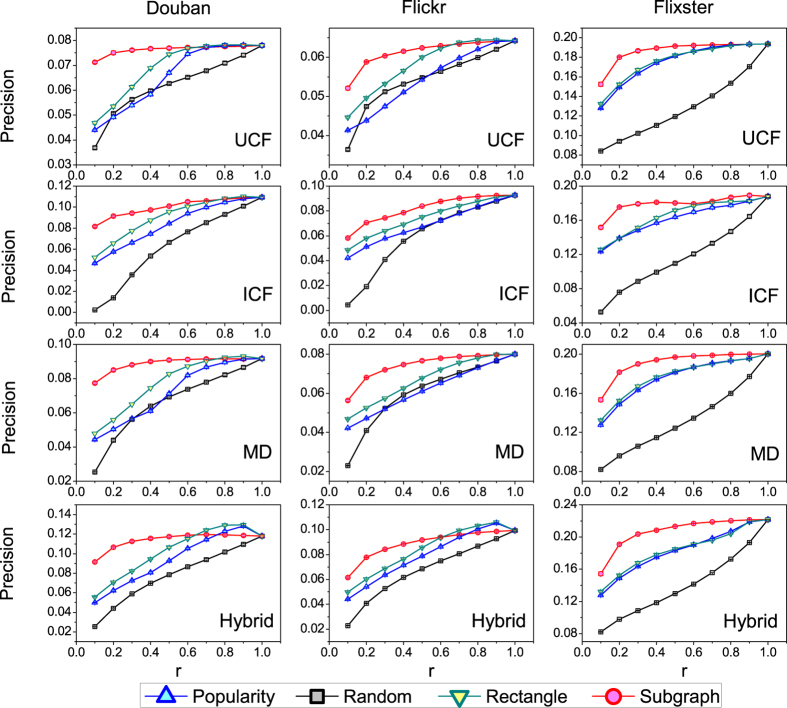
The recommendation accuracy contributed only by the backbone in recommender system. The recommendation length *L* is set to 20. For the subgraph-based method, the number of neighboring users and objects is set to 10. *r* is the ratio of the size of the backbone to the whole system. The error bars are obtained based on 5 independent instances of training and probe set.

Generally speaking, the more data we used in a recommender system, the higher accuracy we can get. In this paper, our purpose is to find the most relevant links in the user-object bipartite network, with which a recommendation algorithm can achieve a satisfactory accuracy. The result is presented in Fig. 2, where *r* denotes the fraction of links in the information backbone. When *r* = 1, all the links will be used in the recommendation algorithms, equivalent to the traditional method. For the subgraph-based method, the number of neighboring users and objects is set to 10 (see the results with different neighbor size in SI).

**Figure 3:**
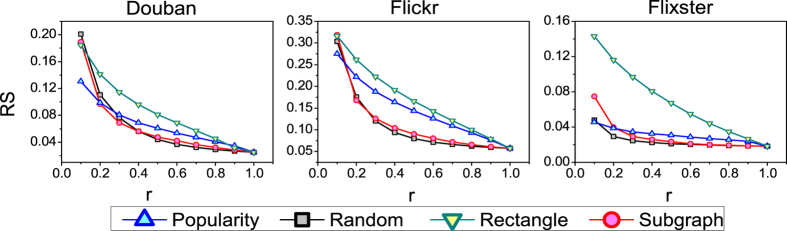
The recommendation accuracy (ranking score) contributed only by the backbone. For the subgraph-based method, the number of neighboring users and objects is set to 10. *r* is the ratio of the size of the backbone to the whole system.

We make use of *precision* index to measure the top-*L* accuracy of the algorithm. Naturally, the accuracy of the recommendation algorithm is lowered if less information is used (*r* < 1). However, the accuracy doesn’t change too much if we use the subgraph-based backbone. Taking the UCF algorithm in the Douban dataset for example, 96.2%(0.075/0.078) of the accuracy can be achieved when we use only 20% links (r = 0.2). It outperforms other methods especially when the size of the information backbone is very small. The similar results can be found in Flickr and Flixster datasets. This is of great importance since the algorithmic efficiency of recommendation methods can be largely improved if we take into account fewer links. In addition to *precision*, we also choose the *ranking score* as the accuracy metric and the results are presented in Fig. 3. To save space, we only plot the result of the UCF recommendation algorithm. It can be seen that recommendation algorithms have the best performance in the backbone extracted by random method. This is because objects selected by the random method are more diverse than other methods and more objects will get resources distributed by users’ selected objects^9^. From practical point of view, online retailers are generally more concerned with the accuracy of the top-L object recommendations. When only the links in the backbone are considered, some users will become isolated, with no adjacent links in the network. The ratio of those isolated users are 4%(480/11898), 3.9%(797/20641), 3.6%(360/10000) for Douban, Flickr and Flixster, respectively. For these users, we recommend the most popular objects.

**Figure 4:**
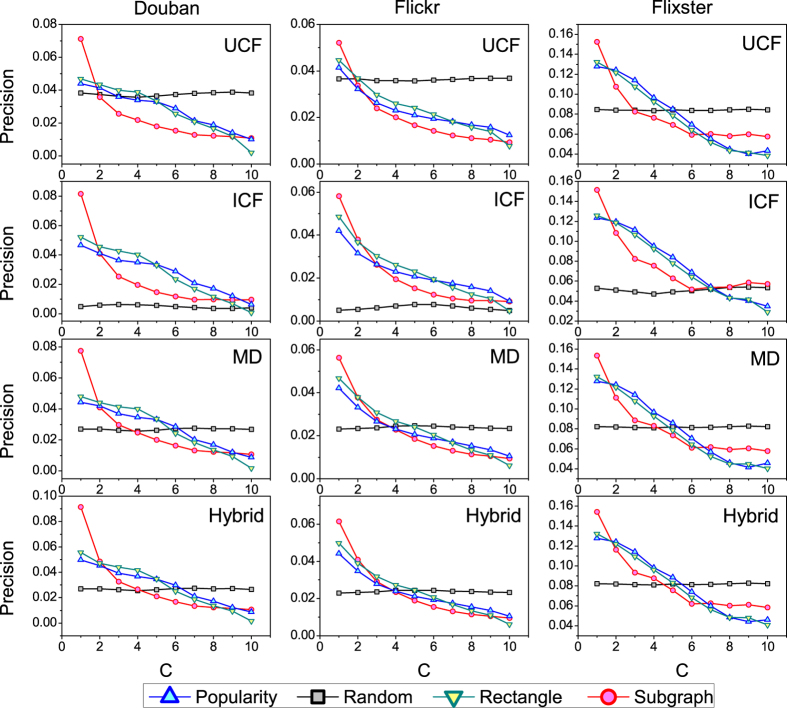
The performance of recommendation algorithms in each subset. For the subgraph-based method, the number of neighboring users and objects is set to 10. The recommendation length L is set to 20.

From the Fig. 2, it can be seen that the random method is sometimes better than the method based on computation (e.g. popularity-based method). In order to uncover the relevance of links in detail and to see their differences, we evenly divide all links into 10 groups *C*_1_, *C*_2_, …, *C*_10_ according to their weights computed by the information backbone extraction methods. If *i* < *j*, the weights of all links in *C*_*i*_ are larger than that in *C*_*j*_. We also choose the above four recommendation algorithms to test the information backbone extraction methods and the results are presented in Fig. 4, where only the links in *C*_*i*_ are used to run the algorithm. Likewise, we make use of the *precision* index to test the accuracy of the algorithms. Firstly, for subgraph-based, popularity-based and rectangle-based methods, the accuracy of recommendation algorithms considering links in group *C*_*i*_ is better than *C*_*j*_ with *i* < *j*, which means all these methods have the ability to uncover the relevant links compared to the random method. Besides, when only the links in *C*_1_ are considered, the subgraph-based method is the best one compared to other methods, which means the relevant links uncovered by subgraph-based method carry the most information of the recommender system.

Moreover, we investigate the structure of the information backbone. For simplicity, the relative size of the information backbone (*r*) is set as 0.2. We then compute the average degree 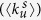 of users and the average degree 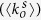 of objects which are selected by users in the information backbone. The result is presented in Table 2. Firstly, 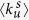 in the rectangle-based information backbone is the largest, which means many large degree users are selected in the backbone. Secondly, the backbone obtained by random method has the smallest 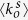 since this method chooses diverse objects. For the graph-based backbone, its objects are less diverse than objects selected by random method, but its 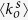 is much smaller than 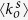 of the popularity-based and rectangle-based backbone.

In addition, we choose another three indices to uncover the structure of the information backbone: degree heterogeneity, clustering coefficient and diffusion coverage. The degree heterogeneity is defined as 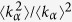 where 〈*k*_*α*_〉 is the average degree of objects. The results are presented in Table 3, which reveals that the subgraph-based backbone has the largest degree heterogeneity, in other words, this method tends to select those high quality objects in the backbone^20^.

For an object *α*, its clustering coefficient is defined as 
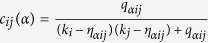
, where *i* and *j* are neighboring users of object *α*, *q*_*αij*_ is the number of common neighbors between *i* and *j* (not counting *α*), *η*_*αij*_ = 1 + *q*_*αij*_. The clustering coefficient of *α* is defined as the average of *c*_*ij*_*α* over all the users. The clustering coefficient of the network is the average clustering coefficient of all the objects^21^. Generally speaking, the backbone has a smaller clustering coefficient since a lot of links are removed. For the Douban and Flickr datasets, the subgraph-based backbone has the largest clustering coefficient, which means the objects in the backbone are highly overlapped. This could explain why the subgraph-based backbone has the best predictive accuracy^22^. For the Flixster dataset, the rectangle-based backbone has the largest clustering coefficient since the degree distribution of object is highly uneven (see Table 2).

The diffusion coverage is defined as the ratio of relevant objects to all the objects in the network^9^. Given a diffusion starting from user *i*, in the first step, it finds the relevant users who have collected the same objects with *i*. In the second step, those objects which are selected by the relevant users are found and marked as relevant objects. From the Table 3, the diffusion coverage of the backbone is much smaller than that of the original network, which means many irrelevant objects are filtered out. For the popularity-based and the rectangle-based backbone, their diffusion coverage is too small, which affects the diversity of a recommendation algorithm. Taking the UCF in the Douban dataset for example, its diversity decreases from 0.5 to 0.1 (see the results in SI).

## Discussion

In summary, this paper proposes an enhanced topology-aware method to uncover the information backbone in a user-object bipartite network. The basic assumption of our method is that the more frequently a link is used, the more relevant it is. We firstly find out the subgraph related to neighboring users and objects, and then count the frequency of a link appearing in these subgraphs. Compared to existing methods, our method utilized more information. By investigating the structure of the backbone, it is found that the backbone extracted by our method has a higher cluster coefficient and lower diffusion coverage. It means the objects in the backbone are highly overlapped and a lot of irrelevant objects are filtered out, which could explain why the subgraph-based backbone has the best predictive accuracy. This work may have wide applications in practice. For one thing, the efficiency of a recommendation algorithm can be significantly improved since a lot of links are removed. Although it takes time to get the information backbone, the backbone is quite relatively stable in real systems. Taking the Douban dataset for example, more than 97% links stay the same in two adjacent months. Therefore, it is enough to update the information backbone once a month, which will significantly reduce the computational cost. For another, it can be very helpful to study topological properties or functions of the original network according to the structure of the backbone.

There are still many open issues. On one hand, we concentrate on the topology-aware backbone extraction method without considering the time factor. A recommender system is actually a evolving system, therefore the information backbone should be changing dynamically. To better understand the backbone, one could study the evolution of the original network and the backbone, respectively. On the other hand, we only remove those irrelevant links in the recommender system. If both the irrelevant links and users are removed simultaneously, the efficiency of the recommendation algorithm can be further improved^10^.

## Methods

### Data description

Douban, launched on March 6, 2005, is a Chinese Web 2.0 website providing user reviews and recommendation services of movies, books, and music^23^. The raw data contains user activities before Aug 2010 and we randomly sample 11,898 users who have collected at least twenty songs. Flixster is a social movie site allowing users to share movie ratings, discover new movies and meet others with similar movie favor^24^. The raw data consists of 147,612 users and we randomly sample 10,000 users who have collected at least twenty movies. Flickr is an image hosting and video hosting website, web services suite and online community^25^. In this paper, data of individuals’ membership on that are concerned. Therefore, we recommend groups for users instead of objects^26^,^27^ and randomly sample 20,641 users who have joined at least twenty groups (see Table 1). As it is quite difficult to provide accurate recommendations for those users with small degree, we filter out the users whose degrees are no larger than 20 in advance.

### Evaluating recommender systems

Each data is randomly divided into two parts: the training set (*E*^*T*^) and the probe set (*E*^*P*^). The training set contains 80% of the original data and the recommendation algorithm runs on it^24^. The probe set contains the remaining 20% links and will be used to test the performance of the recommendation algorithm. In our simulation, we run five independently random divisions of *E*^*T*^ and *E*^*P*^ and average the results^28^.

#### Precision

The precision of a user *i* is defined as *P*_*i*_(*L*) = *d*_*i*_(*L*)/*L*, where *L* is the length of the recommendation list and *d*_*i*_(*L*) is the number of hit links, namely user *i*’s probe set links contained in the top-L recommendation. The precision of the whole system is defined as 
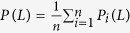
. A higher precision value indicates a better recommendation^29^.

#### Ranking score

We also consider a global accuracy metric: ranking score(RS)^23^. For a target user *i*, the recommendation algorithm will give her uncollected objects a rank and the ranking score is defined as *RS*_*iα*_ = *p*/*N*_*i*_, where *p* is the ranking position of object *α* in the probe set and *N*_*i*_ is the total number of uncollected objects. The lower the ranking score, the better the recommendation. The mean value of RS is defined as 
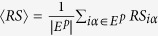
.

### Mass diffuse

It works by assigning objects an initial level of “resource” denoted by the vector 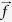 (where *f*_*α*_ is the resource possessed by object *α*), and then redistributing it via the transformation 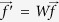, where 
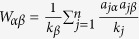
 is a column-normalized *m* × *m* matrix. For a target user, the resulting recommendation list of uncollected objects is sorted according to 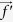 in descending order and top-*L* objects with the most resources will be recommended.

### Hybrid algorithm

When recommending objects for user *i*, the hybrid method works by assigning one unit of resource to each object which is collected by user *i*. The initial resources are denoted by the vector 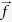 where *f*_*α*_ is the resource possessed by object *α*. Then they will be redistributed via the transformation 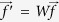, where 
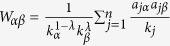
 is the redistribution matrix, with 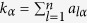 and 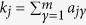 denoting the degree of object *α* and user *j*, respectively. *λ* is a tunable parameter which adjusts the relative weight between the mass diffusion algorithm (*λ* = 1) and heat conduction algorithm (*λ* = 0).

### User-based collaborative filtering

In the user-based collaborative filtering method, the basic assumption is that similar users usually have common interests and collect the same objects. Accordingly, the recommendation score of object *α* for the target user *i* is 
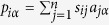
, where *s*_*ij*_ is the cosine similarity between user *i* and *j*.

### Item-based collaborative filtering

It provides each individual user with objects which are similar to her selected objects. That is, for user *i*, the recommendation score of object *α* is 
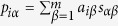
 where *s*_*αβ*_ is the cosine similarity between objects *α* and *β*. Objects will be sorted in descending order according to *p*_*iα*_ and the highly ranked objects will be recommended to *i*.

## Additional Information

**How to cite this article**: Zeng, W. *et al.* Uncovering the essential links in online commercial networks. *Sci. Rep.* **6**, 34292; doi: 10.1038/srep34292 (2016).

## Supplementary Material

Supplementary Information

## Figures and Tables

**Table 1:** The statistics of Douban, Flickr and Flixster datasets.

Dataset	#Users, *n*	#Objects, *m*	#Links, *l*	Sparsity
Douban	11,898	222,815	2,037,736	7.69 × 10^−4^
Flickr	20,641	58,768	1,795,730	1.48 × 10^−3^
Flixster	10,000	40,758	2,106,568	5.17 × 10^−3^

The sparsity is defined as 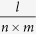.

**Table 2:** The average degrees of users and the average degrees of objects which are selected by these users in the information backbone.

Methods	Douban		Flickr		Flixster	
	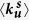	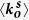	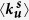	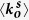	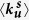	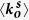
Random	150.33	15.57	71.01	37.44	176.64	84.89
Subgraph	145.50	42.48	72.11	84.67	175.39	318.71
Popularity	413.85	65.48	86.83	184.34	461.66	338.10
Rectangle	392.49	121.08	101.61	236.45	480.21	889.41

**Table 3:** The characteristics of original network and the information backbone.

	Original	Random	Subgraph	Popularity	Rectangle
Degree heterogeneity					
Douban	25.91	10.88	47.44	12.41	6.75
Flickr	14.58	9.69	49.64	16.55	9.78
Flixster	26.00	12.59	26.65	9.14	3.06
Cluster coefficient					
Douban	5.906e-05	2.046e-05	2.237e-05	1.368-e05	1.746e-05
Flickr	9.588e-04	3.107e-04	4.895e-04	1.045e-04	2.011e-04
Flixster	2.391e-04	6.961e-05	2.387e-04	2.304e-04	3.190e-04
Diffusion Coverage					
Douban	0.8470	0.1364	0.1074	0.0211	0.0097
Flickr	0.7723	0.1438	0.1530	0.0572	0.0291
Flixster	0.9911	0.3256	0.1128	0.0313	0.0077
