# Supplementary material for: Uncovering the essential links in online commercial networks
Source: Sci Rep. 2016 Sep 29;6:34292. doi: 10.1038/srep34292 (PMC5041110; doi:10.1038/srep34292)
Supplement: Supplementary Information [file srep34292-s1.pdf]

Supplementary Information

Uncovering the essential links in online commercial networks

Wei Zeng[1,2,3,*], Meiling Fang[1,2,3], Junming Shao [2,3], and Mingsheng Shang[4,*]

[1] *Trusted Computing and Automated Reasoning Lab,*

University of Electronic Science and Technology of China, Chengdu 611731, P.R. China

[2] *Web Sciences Center, University of Electronic Science*

and Technology of China, Chengdu 611731, P.R. China

[3] *Big Data Research Center, University of Electronic*

Science and Technology of China, Chengdu, China

[4] *Chongqing Institute of Green and Intelligent Technology, Chongqing 400714, China*

* *Correspondence should be addressed to zwei504@uestc.edu.cn, msshang@cigit.ac.cn*

1. The accuracy of the recommendation algorithm

Apart from the *precision* metric, we also make use of *recall* to measure the accuracy of recommendation algorithms. A brief description of recall metric is shown as follows:

Recall — The recall of recommendation to user i is defined as $R_i(L) = d_i(L)/E_i$, where E_i denotes the number of $u_i's$ links in the probe set and we calculate $d_i(L)$ as the number of her items in the probe set which appear in the recommendation list. Similarly, the recall of the whole system is defined as $R(L) = \frac{1}{n}\sum_{i=1}^{n} R_i(L)$. Higher precision and recall indicate higher accuracy of recommendations.

We choose the UCF recommendation algorithm to test the contribution of the backbone and the results on the metric of *Precision* and *Recall* are presented in Fig. S1. It can be seen that the plot of *recall* is quite similar to the plot of *precision* since their definitions are very similar to each other. For the recall metric, the subgraph-based method also enables the recommendation algorithm to achieve more than 90% of the accuracy of the top-L recommendation while consuming only 20% links.

2. The diversity of the recommendation algorithm

In this section, we study the diversity of the user-based recommendation algorithm with different size of backbone, namely Hamming Distance. The definition is shown as follows:

Hamming Distance — This metric considers the uniqueness of different users' recommendation list. Given two users i and j, the difference between their recommendation lists can be $H_{ij}(L) = 1 - C_{ij}(L)/L$, where C_{ij} is the number of common items in the *top-L* places of both lists. Clearly, if user i and j have the same list, $H_{ij}(L) = 0$, while if their lists are completely different, $H_{ij}(L) = 1$. Averaging $H_{ij}(L)$ over all user pairs we obtain the mean distance $H(L)$, for which greater or lesser values mean, respectively, greater or lesser personalization of users' recommendation lists.

We choose the UCF as the test method and the result is presented in figure S2. For the subgraph-based backbone, the diversity of UCF doesnt change too much since the backbone preserves the topological properties or the function of the original network. For both the popularity-based and rectangle-based backbone, the diversity of UCF declines greatly, which means these backbones reinforce the rich-gets-richer effect in the recommendation process. If we randomly select a part of links in the backbone, it increases the diversity of UCF.

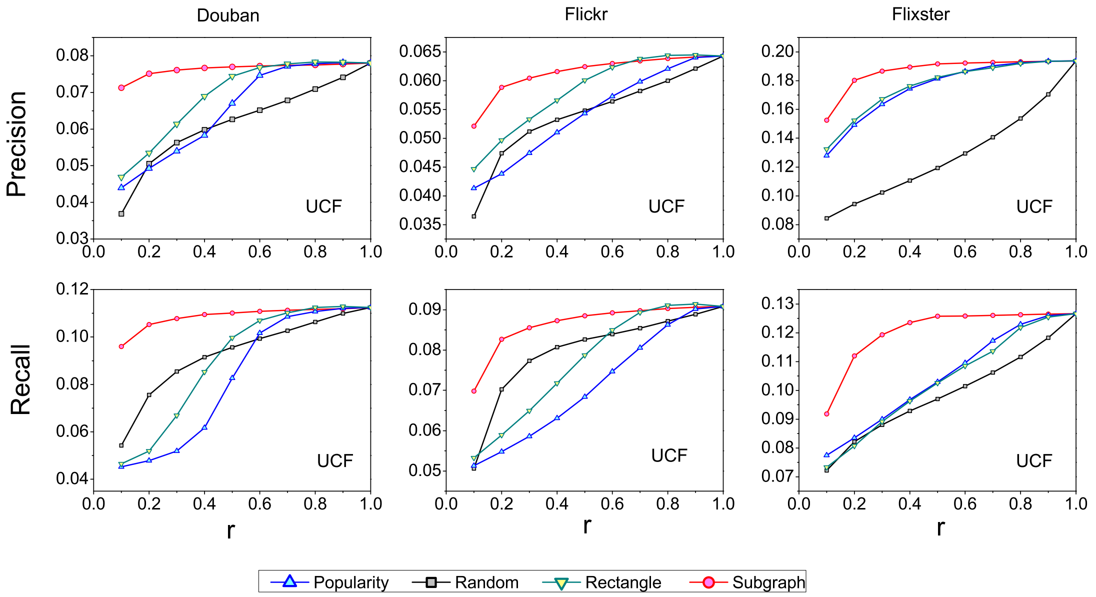

Figure S1 — The UCF's accuracy contributed only by the backbone on the metric of *Precision* and *Recall*. The recommendation length L is set to 20. For the subgraph-based method, we select each users' top-10 neighbours. r is the ratio of the size of the backbone to the whole system. The error bars are obtained based on 5 independent instances of training and probe set.

However, its a little different for the Flixster dataset, in which the diversity of UCF decreases firstly and then increases. The possible reason could be that the object degree distribution is too uneven (See table 2 and 3 in MS).

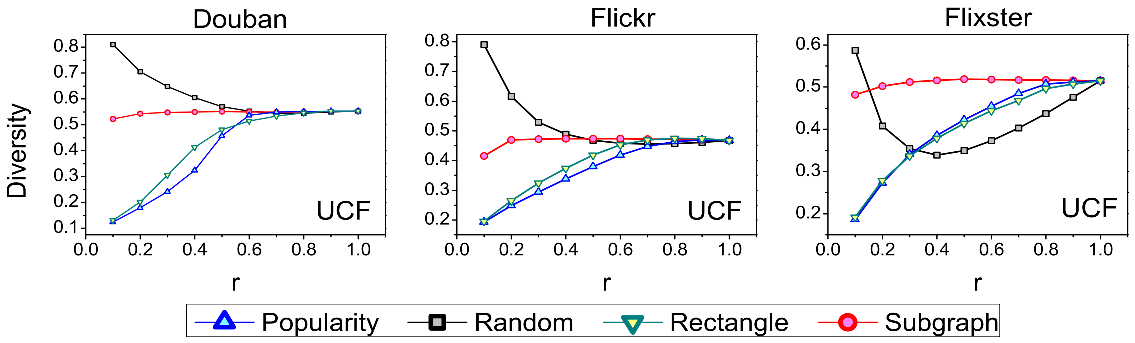

Figure S2 — The UCF's diversity contributed only by the backbone. The recommendation length L is set to 20. For the subgraph-based method, we select each users' top-10 neighbours. r is the ratio of the size of the backbone to the whole system. The error bars are obtained based on 5 independent instances of training and probe set.

3. The neighbor size of the graph-based backbone

In the manuscript, we choose top-10 neighboring users and objects in the subgraph-based backbone extraction method. In this section, the neighbor size is set to 10, 20 and 50, respectively. The UCF algorithm is chosen to test the method and the result is presented in Fig. S3. From the figure, it can be seen that the accuracy of the UCF doesnt change so much when the size of backbone r exceeds 0.2.

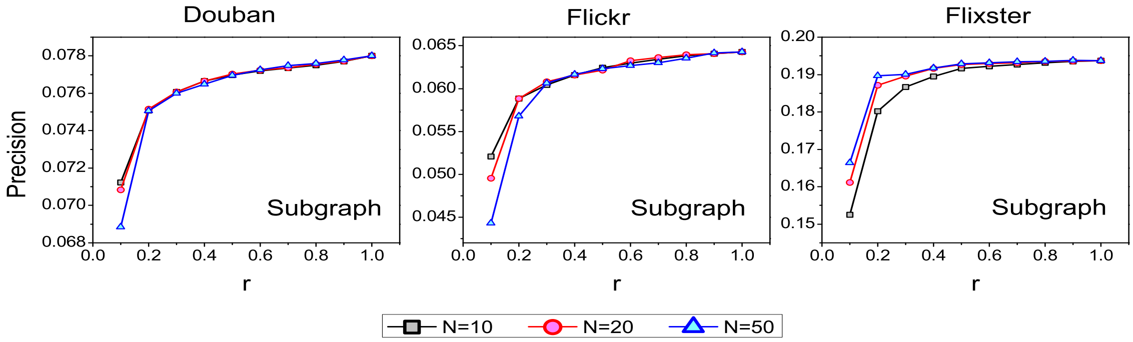

Figure S3 — The neighbor size of the subgraph-based method. The number of neighboring users and items in the graph-based method are set to 10, 20 and 50, respectively. The recommendation length L is set to 20. r is the ratio of the size of the backbone to the whole system.

Table S1. The optimal parameter of hybrid algorithms in the Fig.2 of the main manuscript

Douban

Methods	Random	Similarity	Popularity	Rectangle
size=10	1.0	0.2	0	0
size=20	1.0	0.3	0	0
size=30	0.9	0.3	0	0
size=40	0.8	0.3	0	0.1
size=50	0.7	0.4	0	0.1
size=60	0.6	0.4	0.1	0.2
size=70	0.6	0.4	0.2	0.2
size=80	0.5	0.4	0.2	0.2
size=90	0.5	0.4	0.2	0.3
size=100	0.4	0.4	0.4	0.4

Flickr

Methods	Random	Similarity	Popularity	Rectangle
size=10	1.0	0.2	0.1	0
size=20	1.0	0.2	0.1	0.1
size=30	0.9	0.3	0.1	0.1
size=40	0.8	0.3	0.1	0.1
size=50	0.7	0.3	0.1	0.2
size=60	0.6	0.4	0.2	0.2
size=70	0.6	0.4	0.2	0.2
size=80	0.5	0.4	0.2	0.3
size=90	0.5	0.4	0.2	0.3
size=100	0.4	0.4	0.4	0.4

Flixster

Methods	Random	Similarity	Popularity	Rectangle
size=10	1.0	0.1	1.0	0.9
size=20	0.8	0.1	1.0	0.4
size=30	0.6	0.1	0.5	0.1
size=40	0.5	0.2	0.2	0.1
size=50	0.4	0.2	0.1	0.1
size=60	0.4	0.2	0	0.1

Table S2. The optimal parameter of hybrid algorithms in the Fig.4

		Douban		
Methods	Random	Similarity	Popularity	Rectangle
C_1	1.0	0.2	0	0
C_2	1.0	0.4	0.3	0.2
C_3	1.0	0.6	0.4	0.3
C_4	1.0	0.7	0.4	0.4
C_5	1.0	0.7	0.5	0.6
C_6	1.0	0.8	0.6	0.7
C_7	1.0	0.8	0.6	0.8
C_8	1.0	0.9	0.8	0.9
C_9	1.0	0.9	0.8	1.0
C_{10}	1.0	0.9	1.0	1.0
		Flickr		
Methods	Random	Similarity	Popularity	Rectangle
C_1	1.0	0.2	0.1	0
C_2	1.0	0.4	0.3	0.3
C_3	1.0	0.6	0.4	0.4
C_4	1.0	0.8	0.5	0.6
C_5	1.0	0.8	0.6	0.7
C_6	1.0	0.9	0.6	0.8
C_7	1.0	0.9	0.7	0.8
C_8	1.0	0.9	0.7	0.8
C_9	1.0	0.9	0.7	0.9
C_{10}	1.0	0.8	1.0	1.0
		Flixster		
Methods	Random	Similarity	Popularity	Rectangle
C_1	1.0	0.1	1.0	0.9
C_2	1.0	0.2	0.9	0.9
C_3	1.0	0.3	0.8	0.2
C_4	1.0	0.4	0.2	0.1
C_5	1.0	0.4	0.2	0.2
C_6	1.0	0.5	0.2	0.2
